# Characteristic and Functional Study of *Intersex*, a Gene Related to Female Fertility in *Bemisia tabaci*

**DOI:** 10.3389/fphys.2020.00055

**Published:** 2020-02-25

**Authors:** Yating Liu, Jinjian Yang, Zhijia Huo, Shaoli Wang, Qingjun Wu, Xuguo Zhou, Wen Xie, Youjun Zhang

**Affiliations:** ^1^Department of Plant Protection, Institute of Vegetables and Flowers, Chinese Academy of Agricultural Sciences, Beijing, China; ^2^Department of Entomology, University of Kentucky, Lexington, KY, United States

**Keywords:** *Bemisia tabaci*, *intersex*, RNA interference, sex determination, pest control

## Abstract

The *intersex* (*ix*) gene acts in concert with *doublesex* (*dsx*) at the end of the sex determination hierarchy to control somatic sexual differentiation in *Drosophila melanogaster*. Here, we report the *Drosophila ix* homolog in *Bemisia tabaci* (*Btix*) with differential splicing events. Four isoforms were found in *B. tabaci* adults, including two sex-specific transcripts (*Btix*^*F*^ and *Btix*^*M*^). Knockdown of *Btix* had no measurable effects on female morphological phenotypes but reduced the expression of the *vitellogenin* gene and resulted in the production of significantly fewer eggs, a lower eclosion rate, and a shorter body size of female progeny in comparison with control females. These results increase our understanding of the genes underlying sex determination in *B. tabaci* and reveal a potential target for RNA interference-based pest management.

## Introduction

Insect sex determination, which affects the development and reproduction of insects, is the result of long-term natural selection. Through long-term natural selection, insects have developed a great variety of sex determination mechanisms. Even in closely related species, the primary signals are different, for example, dosage of the X-signaling element (XSE) in *Drosophila melanogaster* (Erickson and Quintero, 2007), maternal input of *transformer* (*tra*) messenger RNA (mRNA) in *Ceratitis capitata* (Pane et al., 2002), and also in *Musca domestica* (Hediger et al., 2010) and *Nasonia vitripennis* (Verhulst et al., 2010), heterozygosity of the *complementary sex determiner* (*csd*) locus in *Apis mellifera* (Beye et al., 2003), female-specific *piRNA* in *Bombyx mori* (Kiuchi et al., 2014), a dominant male-determining factor (M factor), the *Nix* gene in *Aedes aegypti* (Hall et al., 2015), the *Guy1* gene in *Anopheles stephensi* (Criscione et al., 2016), the *Yob* gene in *Anopheles gambiae* (Krzywinska et al., 2016), the *Mdmd* gene in *M. domestica* (Sharma et al., 2017), the *MoY* gene in *Ceratitis capitate*, and the *MoY* orthologs in *Bactrocera oleae* and *Bactrocera dorsalis* (Meccariello et al., 2019). Nevertheless, recent studies have indicated that some of the downstream sex determination genes are generally conserved, such as *doublesex* (*dsx*) and *intersex* (*ix*) (Sánchez, 2008).

*Dsx*, a downstream effector of the sex determination cascade, produces male- or female-specific proteins via sex-specific splicing, controlling sexually dimorphic characters in different insect species (Shukla and Nagaraju, 2010). *Ix*, a gene required for female sexual development in *Drosophila*, acts together with *dsx* to regulate terminal differentiation. The gene *ix* has been characterized in *Drosophila virilis*, *Megaselia scalaris*, *B. mori*, *Oncopeltus fasciatus*, and *Cyclommatus metallifer* (Garrett-Engele et al., 2002; Sánchez, 2008; Aspiras et al., 2011; Gotoh et al., 2016). In *D. melanogaster*, *ix* is intronless and produce only one mRNA in all tissues of both males and females (Garrett-Engele et al., 2002). However, in *Maruca vitrata* and *B. mori*, *ix* is a multiexon gene with alternative splicing (AS) (Cavaliere et al., 2009; Arunkumar and Nagaraju, 2011). *Dmix Bmix* and *Mvix* mRNAs encode highly related and similarly sized proteins. Basic Local Alignment Search Tool (BLAST) analysis revealed 71–74% similarity among these three proteins. The IX proteins exhibit conserved organization at the amino (N)-terminal region similar to known transcriptional activation domains (Garrett-Engele et al., 2002). Functional conservation of *ix* has been demonstrated through heterologous expression of other Diptera and Lepidoptera *ix* homologs in transgenic *D. melanogaster* (Siegal and Baker, 2005; Cavaliere et al., 2009). RNA interference (RNAi) experiments with *O. fasciatus* (Hemiptera) and *C. metallifer* (Coleoptera) also indicated that *ix* has a conserved role in the sex determination pathway (Aspiras et al., 2011; Gotoh et al., 2016).

RNAi is a posttranscriptional gene-silencing mechanism triggered by the introduction of double-stranded RNAs (dsRNAs) and is ubiquitous in many eukaryotes, including insects (Wilson and Doudna, 2013). RNAi has become an effective technique for studying gene function in insects and provides a new method for insect pest control. The RNAi-mediated insect pest management strategy is environment friendly and highly specific. It has great application potential in both species-specific biopesticides and transgenic plants (Burand and Hunter, 2013).

The whitefly *Bemisia tabaci* (Hemiptera: Aleyrodidae) is a worldwide agricultural pest with a haplodiploid reproductive system (Blackman and Cahill, 1998). Our laboratory previously identified 26 genes putatively associated with sex determination in *B. tabaci* and found that *da*, *mle*, and *PSI* undergo sex-specific splicing in *B. tabaci*, while *Imp*, *dsx*, and *tra2* do not (Liu et al., 2016, 2019; Guo et al., 2018). Using RNAi experiments, we also found that *Btdsx* and *Bttra2* affect each other and are important for male genitalia formation (Guo et al., 2018). *Btdsx* regulates the expression of *Vg* in *B. tabaci* females (Guo et al., 2018). *Bttra* directly regulates the expression of *Btfru* and *Btdsx*. *Bttra* and *Btdsx* affect each other, and autoregulation of *tra* may exist in *B. tabaci* (unpublished). In this study, we cloned and characterized the *ix* gene in *B. tabaci*, explored the AS events of *Btix* in adult insects, and investigated the functions in regulating *Vg* expression and reducing female fecundity. The results suggest the potential of *Btix* as a target for RNAi-based pest management.

## Experimental Procedures

### Insect Strains

The Q *B. tabaci* (Mediterranean, MED) population was originally collected from the fields in Beijing, China, in 2009, and was reared on cotton plants (*Gossypium herbaceum* L. cv. Zhongmian 49) in a glasshouse under natural light after isolation. The purity of the strain was monitored every two to three generations using a mitochondrial cytochrome oxidase I (*mtCOI*) marker (Chu et al., 2005). Samples of newly emerged adult females and males (within 1 h) were separately collected using a glass tube (5.0 × 0.5 cm), and the sex of each individual was determined with a stereomicroscope.

### Gene Cloning and Identification of Alternative Splicing Variants

The annotated sequences obtained from our previous study (Liu et al., 2019) were used to design full-length primers to amplify the *Btix* gene (Table 1). The PCRs were performed using Es-Taq Master Mix (CWBIO, Beijing, China) under the following conditions: initial denaturation at 95°C for 5 min, followed by 35 cycles of 95°C for 30 s, 55°C for 30 s, and 72°C for 1 min, and a final extension at 72°C for 10 min. The products were purified by a Monarch DNA Gel Extraction Kit (NEB, Beijing, China), cloned into the pEASY-T1 vector (TransGen, Beijing, China), and sequenced. For AS analysis, complementary DNA (cDNA) from newly emerged female or male samples was used. The products were purified, cloned, and sequenced as described previously.

**TABLE 1:** List of primers used in the experiment.

Application of primers	Gene name	Primer name	Primer sequence (5′–3′)
Cloning, AS analysis	*ix*	Full-F	AATCACTGTTCACCGCTCTT
		Full-R	GGGAGAAGTCGTGAGGGA
qPCR analysis	*ix*	ix-qF5	TATCAATCACTGTTCACCGCTCTT
		ix-qR5	CGGCATACCAGGGGGCAT
	*Vg*	Vg-qF	CAGCAGCGAAGAGGACTATG
		Vg-qR	TAGCGGATTGGATACTGTTACC
	*SDHA*	SDHA-qF	GCGACTGATTCTTCTCCTGC
		SDHA-qR	TGGTGCCAACAGATTAGGTGC
RNAi	*ix*	dsIX-F5	TAATACGACTCACTATAGGC CAGTTCCTCAAGCTCCATC
		dsIX-R5	TAATACGACTCACTATAGGTTTGGT AACGGCTCAGTCCT
	*EGFP*	dsEGFP-F	TAATACGACTCACTATAGGGA GACAGTGCTTCAGCCGCTAC
		dsEGFP-R	TAATACGACTCACTATAGGGAGA GTTCACCTTGATGCCGTTC

### Phylogenetic Analysis and Sequence Alignment

Sequences were aligned using the program ClustalW with the following default gap penalty parameters: a gap opening of 10 and an extension of 0.2. A neighbor-joining (NJ) tree was constructed using the program MEGA 6.0 with a p-distance model and pairwise deletion of gaps (Tamura et al., 2013). The bootstrap support of tree branches was assessed by resampling amino acid positions 1,000 times.

The open reading frame (ORF) of the target nucleotide sequence was predicted by the ORF Finder tool on the National Center for Biotechnology Information (NCBI) website. The deduced protein domains were determined using the NCBI Conserved Domain Search Service. Molecular weights (MWs) and isoelectric points were analyzed via the ExPASy ProtParam tool. Sequence-similarity analyses were performed with DNAMAN 6.0. The amino acid sequences of IX were aligned using the ClustalW program and then shaded by the ExPASy BOXSHADE tool.

### RNA Isolation

Total RNA was extracted using TRIzol reagent (Invitrogen, Carlsbad, CA, United States) according to the manufacturer’s instructions. RNA was quantified using a NanoDrop 2000 (Thermo Scientific, Wilmington, DE, United States), and integrity was checked with 1% Tris/borate/ethylenediaminetetraacetic acid (EDTA) (TBE) agarose gel electrophoresis. For gene cloning and quantitative PCR (qPCR) analysis, first-strand cDNA was synthesized using 1 μg of total RNA with the PrimeScript^®^ RT reagent kit (TaKaRa Biotech, Kyoto, Japan) following the manufacturer’s recommendations. The synthesized first-strand cDNA was used immediately or stored at −20°C for later use.

### Real-Time Quantitative PCR

Quantitative PCR was performed using SuperReal PreMix Plus (SYBR Green) (Tiangen, Beijing, China) according to the manufacturer’s instructions. Each reaction contained 1 μl of cDNA template, 10 μl of SuperReal PreMix Plus, 0.4 μl of ROX reference dye, and 0.5 μl of specific primers in a total volume of 20 μl. PCR was performed under the following conditions: denaturation at 95°C for 10 min, followed by 40 cycles of 95°C for 15 s, 60°C for 30 s, and 72°C for 30 s. The specific primers are shown in Table 1. Three independent biological replicates were included for each treatment. The results were standardized to the expression level of the *B. tabaci SDHA* gene (Li et al., 2013), and the relative differences in transcript levels were analyzed by the 2^–△△Ct^ method (Livak and Schmittgen, 2001).

### RNA Interference

Primers (Table 1) containing a T7 promoter sequence were designed to amplify dsRNA targeting *Btix*. The dsRNA for enhanced green fluorescent protein (EGFP) was used as the negative control. The primers are listed in Table 1. All dsRNAs were prepared using the T7 Ribomax^TM^ Express RNAi system (Promega, Madison, WI, United States). RNAi was achieved by directly feeding dsRNA to *B. tabaci* adults in a feeding chamber. A 0.20-ml drop of diet solution [5% yeast extract and 30% sucrose (wt/vol)] containing 0.5 μg/μl dsRNA was placed in the chamber (Upadhyay et al., 2011; Yang et al., 2017). Approximately 40 newly emerged females were introduced into an environmental chamber at 25°C under a photoperiod of L14/D10 and a relative humidity (RH) of 70%. Each sample was represented by three technical replicates. After 2 days of feeding, mortality was recorded, and *B. tabaci* specimens were collected. To determine the effects of silencing *Btix* on *Vg* expression and female development, we observed the RNAi females for 3 days. The phenotype of *B. tabaci* was observed by a stereomicroscope (Leica, M205C, Germany), and the expression levels of targeted genes were analyzed by real-time qPCR (RT-qPCR). The detail was as follows: first, ∼1,000 newly emerged *B. tabaci* females were observed to make sure that all individuals were wild type. Second, ∼500 newly emerged females were fed on dsBtix, and the others were fed on dsEGFP. Third, after 2 days of feeding, females were observed immediately or at 24, 48, 72 h with a stereomicroscope, and then were collected for qPCR analysis. Each replicate contained 40 females and 3 replicates per treatment. One-way ANOVA with Tukey’s test (*P* < 0.05) were used to evaluate differences among treatments.

### Fecundity of *B. tabaci*

To determine the effects of silencing *Btix* on *B. tabaci* reproduction, after 2 days of dsRNA feeding, females were placed in a plastic bottle (5.5 cm diameter, 15 cm height) with a cotton seedling (Supplementary Figure S1). Each bottle contained five RNAi females and five newly emerged males (20 replicates per treatment). After 3 days of oviposition, the number of *B. tabaci* individuals in each bottle was determined, and their eggs were counted using a stereomicroscope. *B. tabaci* reproduction was reported as the mean number of eggs per surviving whitefly over 3 days (7 days of oviposition was also measured). The eclosion rates were calculated as the total number of emerging adults divided by the number of eggs in each bottle. Newly emerged progenies (females and males) from each treatment were randomly selected and frozen (approximately 90 females and 90 males per treatment). Thereafter, the randomly selected adults were laid on their back, and their length was measured from the top of their head to the end of their abdomen with the aid of a stereomicroscope (Leica, M205C, Germany). Student’s *t*-test (*P* < 0.05) were used to evaluate differences among treatments. SPSS version 19.0 (SPSS Inc., Chicago, IL, United States) was used for statistical analyses.

## Results

### Cloning and Characterization of *Btix*

**FIGURE 1:**
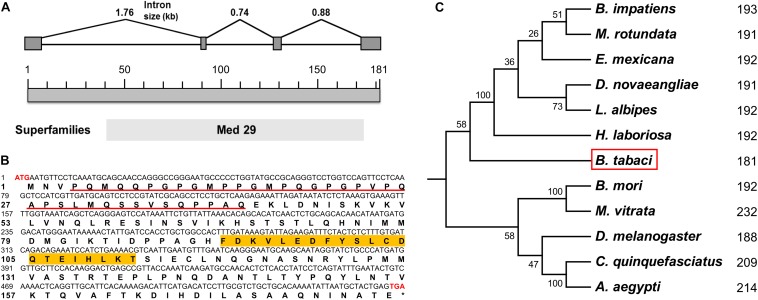
Characteristics of *Btix*. **(A)** Genomic structure of the *Btix* gene in *B. tabaci*. Gray boxes and the spaces between boxes indicate the exons and introns, respectively. The size of the putative exons and introns is drawn to scale. **(B)** Complementary DNA (cDNA) nucleotides and deduced amino acid sequence of the *Btix* gene. The start (ATG) and stop (TGA) codons are highlighted with red bold characters. The glutamine-, glycine-, proline-, and serine-rich regions are underlined, and the DSX^F^ binding region is highlighted in orange. **(C)** Phylogenetic tree of intersex proteins among several insects. Phylogenetic tree of known IX proteins constructed by the neighbor-joining (NJ) method. Each number corresponds to the length of IX for each species.

Based on our previously annotated sequence from the Q/MED *B. tabaci* genome database (Xie et al., 2017; Liu et al., 2019), the full-length cDNA sequence of the *Btix* gene was obtained from PCR amplification, using adult cDNA as a template. The ORF encoded a protein of 181 amino acids with a predicted MW of ∼19.85 kDa and an isoelectric point (pI) of 5.65. In contrast to *ix* in *D. melanogaster* and hymenopterans, which is a single-exon gene, *Btix* possesses four exons (Figure 1A and Supplementary Figure S2).

BtIX contains glutamine-, glycine-, proline-, and serine-rich regions in the N-terminus (corresponding to positions 4–41), and in the C-terminus, there are several stretches of high amino acid identity (with the most striking such block corresponding to residues 92–112), which is a conserved feature of the IX protein (Figure 1B and Supplementary Figure S3).

### Phylogenetic Analysis of *Btix*

We identified putative homologs of IX in three dipteran, two lepidopteran, and six hymenopteran species by searching the BeeBase (Elsik et al., 2016) or NCBI website. Protein sequences were aligned, and an NJ phylogenetic tree was constructed. The resulting tree clustered the IX proteins within each insect order, and BtIX was closely related to the IX from Hymenoptera (Figure 1C).

### Splice Variation of *Btix*

**FIGURE 2:**
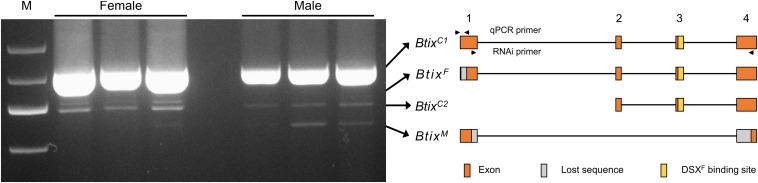
Alternative splicing (AS) analysis of *Btix*. Photograph of an electrophoretic gel showing real-time PCR (RT-PCR) amplification of *Btix* of adult male and female total messenger RNA (mRNA) samples. Structural diagrams of the four *Btix* isoforms found in *B. tabaci* adults are shown on the right. *Btix*^*C*^, non-sex-specific isoform; *Btix*^*F*^, female-specific isoform; *Btix*^*M*^, male-specific isoform. Orange boxes: exons; gray boxes: lost sequences; yellow boxes: DSX^F^ binding sites.

To analyze splice variations of *Btix*, we amplified mRNA products from newly emerged female and male adults using full-length primers (Table 1). A total of four isoforms were found in *B. tabaci* adults: two non-sex-specific isoforms (*Btix*^*C*^), one female-specific isoform (*Btix*^*F*^), and one male-specific isoform (*Btix*^*M*^) (Figure 2). The four isoforms encoded four proteins with different sizes, and among them, only *Btix*^*M*^ encoded a truncated non-functional protein (Supplementary Figure S4).

### *Btix* RNA Interference

**FIGURE 3:**
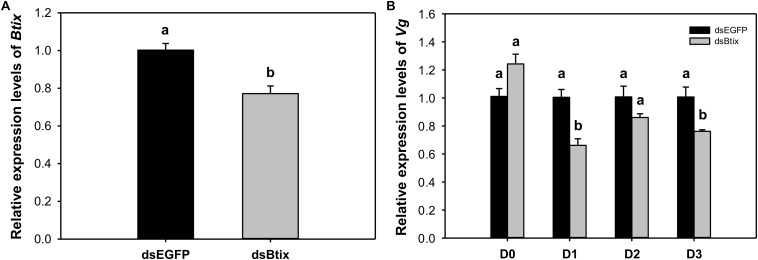
**(A)** The efficiency of RNA interference (RNAi) of *Btix* on newly emerged female adults. **(B)** Relative transcript levels of *Vg* in control and *Btix* double-stranded RNA (dsRNA)-fed females. Bars with the same letter are not significantly different from each other at *P* < 0.05 based on Tukey’s test. Each point represents the mean ± SE from three independent experiments.

To determine the function of *Btix*, we used RNAi technology to knock down the expression of *Btix* in the newly emerged female adults of *B. tabaci*. For dsRNA feeding, RT-qPCR results showed that *Btix* expression was significantly decreased at 2 days of feeding (77.2%, *P* < 0.01; Figure 3A), and the survival rate was more than 95% (Supplementary Figure S5). Furthermore, over the next 3 days, the *Vg* expression in the dsRNA-fed group decreased to varying degrees, particularly on the first (*P* < 0.01) and 3rd days (*P* = 0.018) after RNAi (Figure 3B). After 3 days of observation, no phenotypic (body color and morphological defects of genitalia) changes were observed compared with control females (Supplementary Figure S6).

### Function of *Btix* in Female Reproduction

**FIGURE 4:**
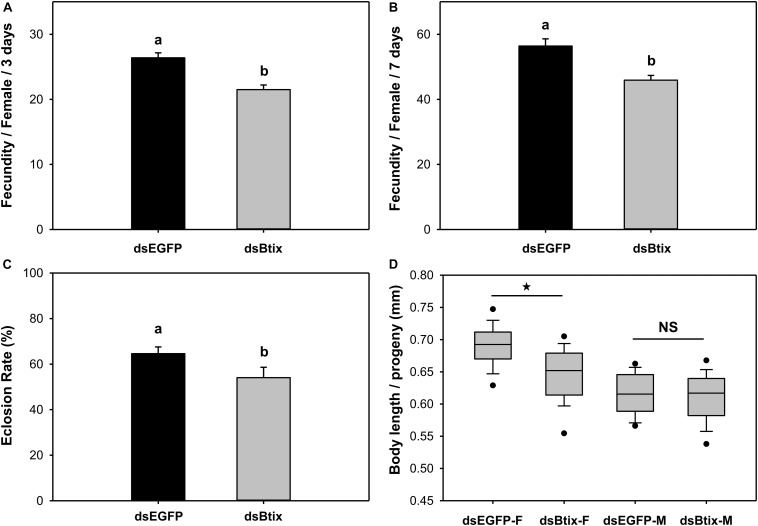
Effect of RNA interference (RNAi) of *Btix* on female reproductive physiology, including fecundity **(A,B)**, eclosion rate of progeny **(C)**, and the body length of progeny **(D)**. Values shown in the figure are means and standard errors. Different letters indicate significant differences between treatments (*P* < 0.05; Student’s *t*-test). The box shows the 25th to the 75th quartiles, and the dark line in the box represents the median value; circles show outliers (5th/95th percentile).

**FIGURE 5:**
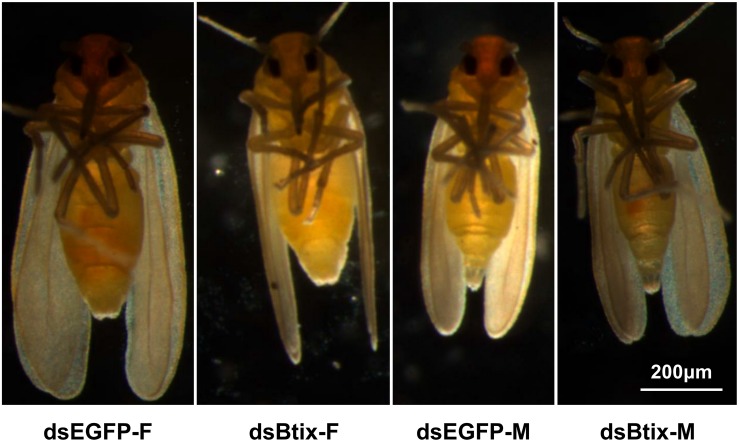
Progeny phenotypes of Btix knockdown.

We found that knocking down the expression of the *Btix* gene affected female reproduction in *B. tabaci*. In *Btix* dsRNA-fed females, fecundity was significantly reduced by 19% (*P* < 0.01; Figures 4A,B), and the eclosion rate of progeny was reduced by 16% (*P* = 0.025; Figure 4C). The average body length of female progeny was also markedly shorter than that of control females (shortened by 7%) (*P* < 0.01; Figures 4D, 5).

## Discussion

In this study, we cloned and characterized the *ix* gene in *B. tabaci* (*Btix*; Figures 1A,B) and found that *Btix* is structurally conserved. First, BtIX shared several modular elements with other insect IX proteins, including the amino acid composition of the N-terminus and high amino acid identity in the C-terminus (Figure 1B and Supplementary Figure S3). The former region resembles known transcriptional activation domains (Garrett-Engele et al., 2002), and the latter is considered an important region for DSX^F^ binding (Yang et al., 2008). Second, the IX homologs were conserved in their short length. BtIX is only 181 amino acids long, and the *D. melanogaster* IX is 188 amino acids in length. The *M. vitrata* IX presents an additional region of 38 amino acids in the C-terminus (Cavaliere et al., 2009) and is the longest homolog, yet it is only 232 amino acids in length (Figure 1C).

*ix* is a single-exon gene and not sex-specifically spliced in *Drosophila* (Garrett-Engele et al., 2002). However, in *M. vitrata* and *B. mori*, the *ix* orthologs present, respectively, five and six exons (Cavaliere et al., 2009). A female-specific and pupae-stage specific *ix* transcript is found in *M. vitrata*, and a testis-specific splice form of *ix* is found in *B. mori* (Cavaliere et al., 2009; Arunkumar and Nagaraju, 2011). In *B. tabaci*, the *Btix* gene contains four exons; a female-specific isoform (*Btix*^*F*^) and a male-specific isoform (*Btix*^*M*^) were found in *B. tabaci* adults (Figure 2). However, we did not find any sex-specific exons, so we were unable to design specific qPCR primers to reveal the developmental and temporal expression patterns of the four isoforms. In addition, since *Btix*^*M*^ only has 186 bp, we did not find a suitable RNAi fragment. Therefore, we only investigated the function of *Btix*^*F*^ mainly in this paper.

The gene *ix* is a conserved downstream gene at the end of the sex determination cascade, second to *dsx*. In *D. melanogaster*, IX specifically interacts with DSX^F^, forming a DNA-binding complex to activate Yolk protein (*Yp*) gene expression and control female sexual differentiation (Garrett-Engele et al., 2002). Female *ix* mutants of *D. melanogaster* display characteristic morphological defects that are highly similar to those in *dsx* mutants (Chase and Baker, 1995; Garrett-Engele et al., 2002). Ectopic expression of *D. virilis* and *M. scalaris ix* cDNA fully rescues the *D. melanogaster* female *ix* mutants, and expression of *B. mori* and *M. vitrata* cDNA partially restores the mutants (Siegal and Baker, 2005; Cavaliere et al., 2009). Knockdown of *ix* in *O. fasciatus* produced defects in both sexes, reducing the size of both male and female genitalia (Aspiras et al., 2011). Depletion of *ix* in *C. metallifer* affected sex-specific morphological characters such as mandible size and body color in females only (Gotoh et al., 2016). These results highlight the conserved function of *ix* in insect sex determination. However, silencing *Btix* did not affect the morphological changes in *B. tabaci* females (Supplementary Figure S6). This may be because we treated the adults. Our lab is working on a new RNAi method for *B. tabaci* nymph and egg stage. When the method is mature, the role of *Btix* in sex determination will be better studied.

In this paper, the expression of *Vg* decreased significantly on the 1st and 3rd day after silencing *Btix* (Figure 3B). This result indicates that *Btix* plays an important role in regulating *Vg* expression. However, the detection of *Vg* expression immediately after RNAi had no effect. This may be because *Vg* is not a target gene for *Btix*. In *D. melanogaster*, yolk protein genes (*Yp1* and *Yp2*) are the best characterized target genes of sex-specific DSX proteins and are affected by any change in the sex-determination pathway (Shukla and Nagaraju, 2010). DSX^F^ regulates *Yp* expression and requires IX; similarly, the female-specific function of IX is mediated by DSX^F^ (Waterbury et al., 1999; Garrett-Engele et al., 2002). To understand the interaction between *Btix* and *Btdsx*, we silenced *Btdsx* and *Btix*, respectively, to detect the expression of these two genes. The results showed that silencing *Btdsx* did not affect the expression of *Btix* and vice versa (Supplementary Figure S7). Since *ix* functions as a complex with DSX in *Drosophila*, the RNAi results can only indicate that there is no cascade control between *Btix* and *Btdsx*. Whether *Btix* combined with *Btdsx* participate in sex determination remain further explained by subsequent experiments.

The *Vg* gene plays a vital role in oocyte and embryo development in insects. Silencing of *vitellogenin* or *vitellogenin receptor* genes resulted in significant mortality and reduced fecundity in *B. tabaci* adults (Upadhyay et al., 2016; Grover et al., 2018). Therefore, it is reasonable to believe that *Btix* may affect reproduction in *B. tabaci*. In the present study, disruption of the expression of *Btix* in female adults reduced fecundity, eclosion rate of progeny, and shortened the body length of female progeny (Figure 4). These results suggest that *Btix* does have an effect on *B. tabaci* reproduction. In previous transgenic *D. melanogaster* tests, female *ix* mutants were sterile; despite expression of the *B. mori ix*, fertility was severely reduced (Siegal and Baker, 2005). These results showed that *ix* has an evolutionarily conserved function in the control of female fertility.

RNAi technology is promising for the control of insect pests; its main challenge is to find suitable target genes. In general, functional genes encoding essential proteins can be suitable RNAi targets. Downregulation of the expression of these genes can reduce the fitness or survival rate of insect pests (Katoch et al., 2013). For example, RNAi knockdown of the P450 *CYP6CM1*, GST, or *HOT* gene increased mortality in a *B. tabaci*-resistant strain (Grover et al., 2018). Knockdown of *SexiCSP3* in *Spodoptera exigua* caused high mortality, low hatching rate, and reduced oviposition (Gong et al., 2012). Knockdown of genes in the ecdysone pathway reduced the survival and delayed the development of *B. tabaci* during nymphal stages (Luan et al., 2013). In this paper, we confirmed the important role of *Btix* in *B. tabaci* reproduction, indicating that *Btix* can be used as a potential target for RNAi-based pest management.

## Conclusion

In the present study, we cloned and characterized the *Btix* gene from *B. tabaci* and provided evidence for the presence of alternatively spliced forms of *ix* in *B. tabaci*. Furthermore, silencing *Btix* downregulated *Vg* expression and had some negative effects on female reproduction in *B. tabaci*. Our findings indicate that *ix* is functionally conserved in the control of female fertility. In addition to expanding our understanding of the genes that may determine sex in *B. tabaci*, these results will help us develop new methods for the effective control of *B. tabaci*. The functional relationships of *Btix* and *Btdsx*, as well as the specific contribution of *Btix* in regulating the terminal differentiation of *B. tabaci*, remain to be further studied.

## Data Availability Statement

The datasets generated for this study can be found in the GenBank: QAB02863.1.

## Author Contributions

All authors listed have made a substantial, direct and intellectual contribution to the work, and approved it for publication.

## Conflict of Interest

The authors declare that the research was conducted in the absence of any commercial or financial relationships that could be construed as a potential conflict of interest.
