# Supplementary material for: Characteristic and Functional Study of Intersex, a Gene Related to Female Fertility in Bemisia tabaci
Source: Front Physiol. 2020 Feb 25;11:55. doi: 10.3389/fphys.2020.00055 (PMC7052062; doi:10.3389/fphys.2020.00055)
Supplement: Supplementary file 1 [file Data_Sheet_1.pdf]

Supplementary Material

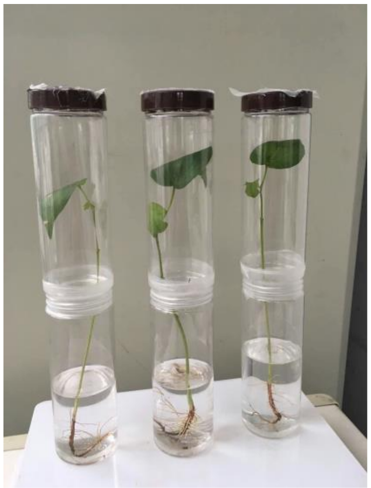

Figure S1. Picture of the plastic bottle device.

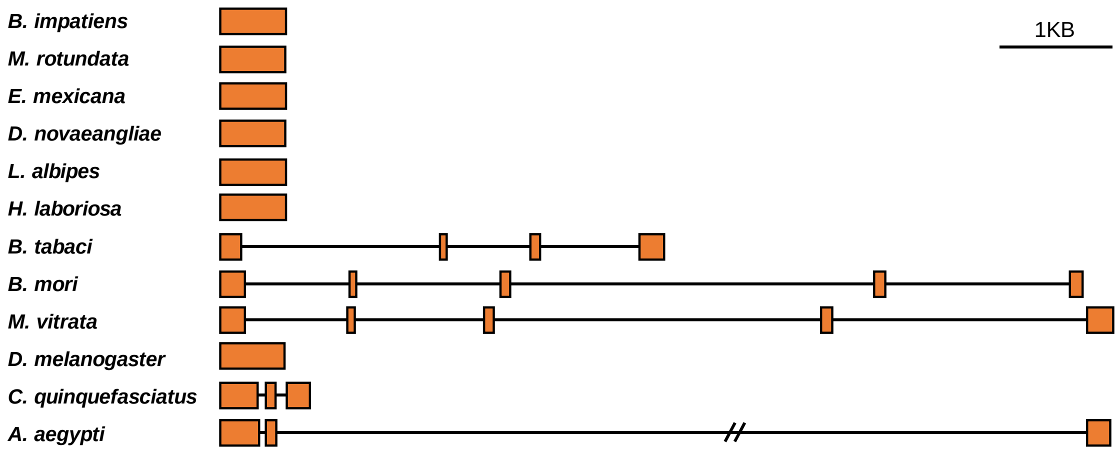

Figure S2. Genomic structure of the *ix* gene for several insects.

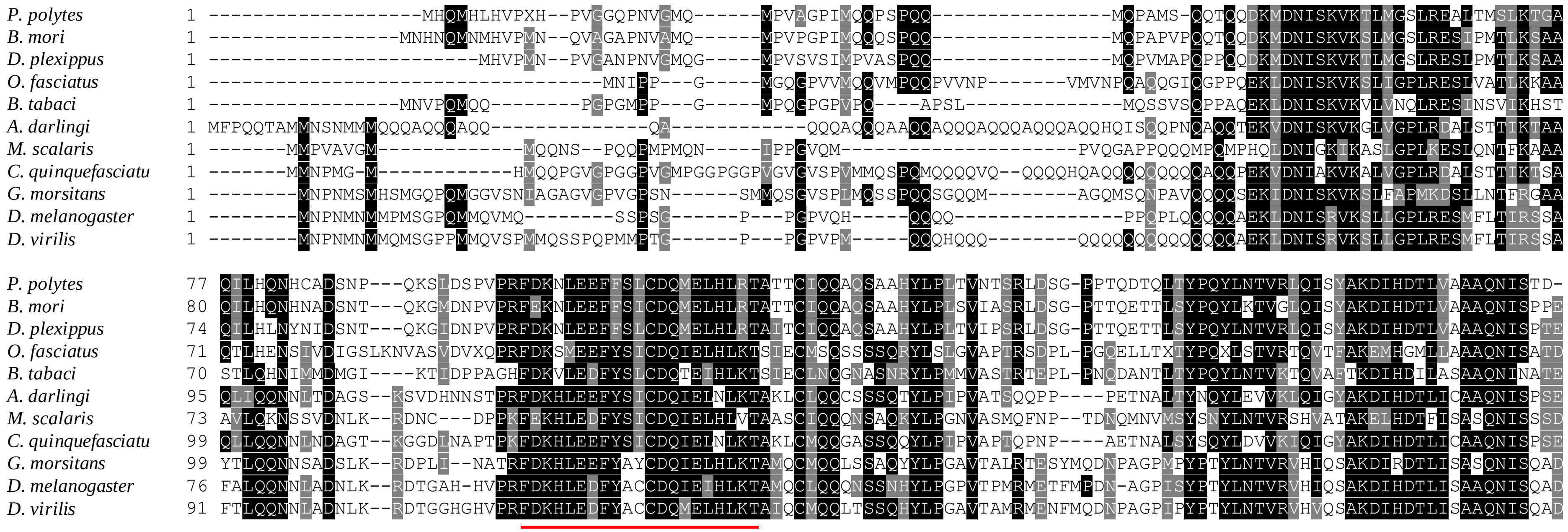

Figure S3. Amino acid alignment of intersex IX homologues. Conserved and similar amino acid residues are indicated as white letters on a black background and white letters on a dark grey background, respectively. Non-conserved residues are indicated as black letters on a white background. The DSX^{F} binding region is underlined.

frontiers

```
BtIXC1  MNVPQMQQPGPGMPPGMPQGPGPVPQAPSLMQSSVSQPPAQEKLDNISKVKVLVNQLRESI
BtIXF   MNVPQ...................................APSLMQSSVSQPPAQEKLDNISKVKVLVNQLRESI
BtIXC2  .............................................................................
BtIXM   MNVPQMQQPGPGMPPGMPQGPGPVPQAPSLMQSSVSQPPAQ....................................
                                                                      Med29
BtIXC1  NSVIKHSTSTLQHNIMMDMGIKTIDPPAGHFDKVLEDFYSLCDQTEIHLKTSIECLNQGN
BtIXF   NSVIKHSTSTLQHNIMMDMGIKTIDPPAGHFDKVLEDFYSLCDQTEIHLKTSIECLNQGN
BtIXC2  ..............................MMDMGIKTIDPPAGHFDKVLEDFYSLCDQTEIHLKTSIECLNQGN
BtIXM   .......................................................................

BtIXC1  ASNRYLPMMVASTRTEPLPNQDANTLTYPQYLNTVKTQVAFTKDIHDILASAAQNINATE
BtIXF   ASNRYLPMMVASTRTEPLPNQDANTLTYPQYLNTVKTQVAFTKDIHDILASAAQNINATE
BtIXC2  ASNRYLPMMVASTRTEPLPNQDANTLTYPQYLNTVKTQVAFTKDIHDILASAAQNINATE
BtIXM   ..........................................................KTFMTSLRLLHKILMLLSEP
```

Figure S4. Amino acid alignments of predicted proteins coded by four *ix* transcripts. The conserved domain (Med29) of IX is highlighted in yellow.

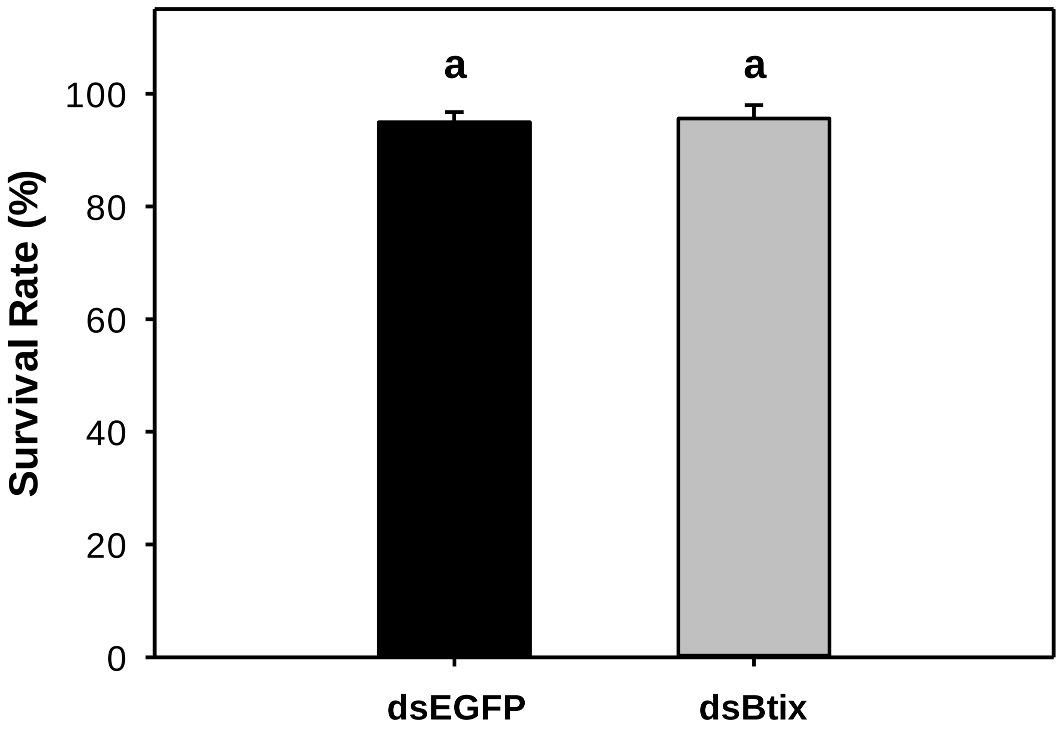

Figure S5. The effect of RNAi of *Btix* on the survival rate of newly emerged female adults. Bars with the same letter are not significantly different from each other at $P<0.05$ based on Tukey's test. Each point represents the mean ± SE from three independent experiments.

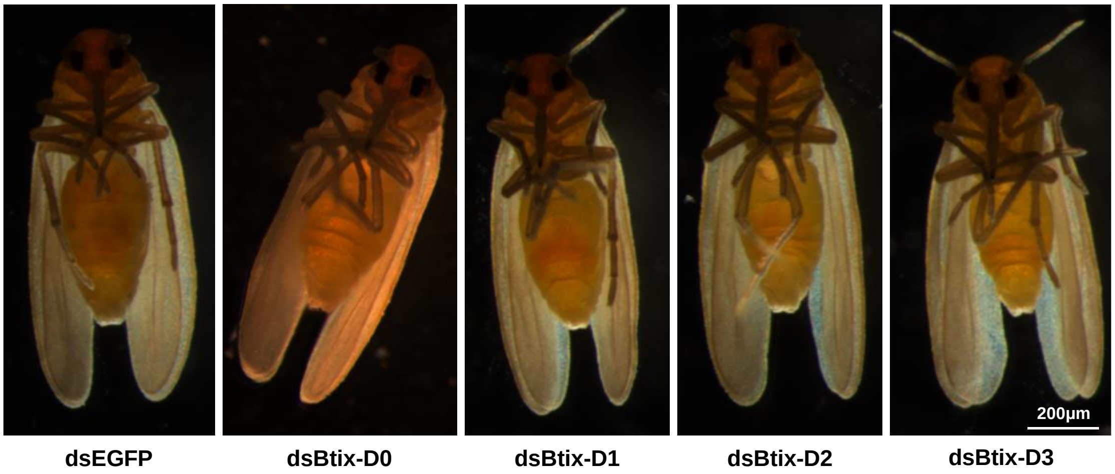

Figure S6. RNAi phenotypes of *Btix*. D0-D3, 0-3 days after RNAi, respectively.

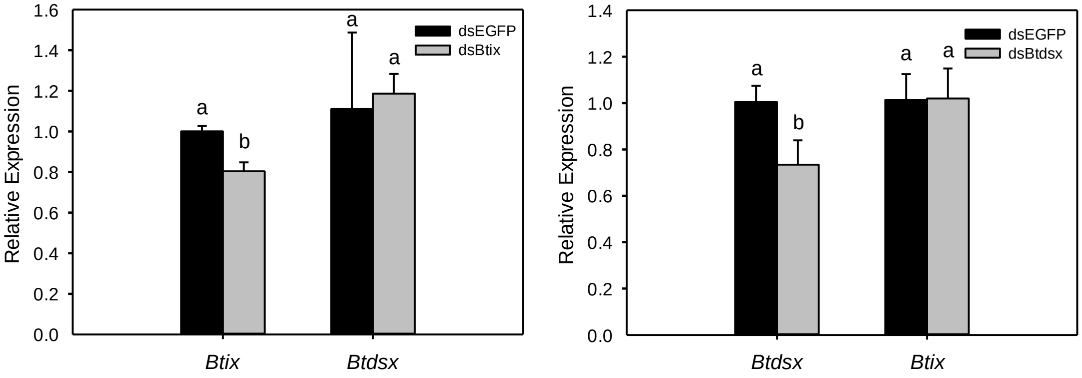

Figure S7. Effect of RNAi *Btix* or *Btdsx* on the expression of *Btix* and *Btdsx*. Bars with the same letter are not significantly different from each other at $P<0.05$ based on Tukey's test. Each point represents the mean ± SE from three independent experiments.
